# Experimental Nursery Schools

**Published:** 1921-01-01

**Authors:** 


### Experimental Nursery Schools.

The London County Council expects much health work to be carried out in connection with the new nursery schools. Some experimental nursery schools are to be shortly established, and the Council proposes that the managers of these new institutions shall be closely associated with the health side of the work, and should also be a link with the ordinary elementary schools. It has therefore been arranged that the managers of each nursery school consist of six persons, two nominated by the managers of the elementary school to which the nursery school is to be attached, two by the Care Committee of the school, and two by the Central Care Committee.

